# The emerging need to integrate digital health literacy as a course into health-related and care-related profession curricula

**DOI:** 10.3389/fpubh.2025.1534139

**Published:** 2025-02-24

**Authors:** Arben Boshnjaku, Ermira Krasniqi, Ferdi Kamberi

**Affiliations:** ^1^Faculty of Medicine, University “Fehmi Agani” in Gjakova, Gjakova, Kosovo; ^2^Faculty of Pharmacy, Alma Mater Europaea Campus College Rezonanca, Prishtina, Kosovo; ^3^Faculty of Social Sciences, University “Fehmi Agani” in Gjakova, Gjakova, Kosovo

**Keywords:** digitalization, digital transformation, health literacy, innovative teaching, healthcare curriculum

## 1 Introduction

As we are rapidly moving toward a predominantly digital age, the health sector faces unprecedented challenges in ensuring that healthcare and other care providers possess at least the necessary digital health literacy (DHL) skills to be in line with the ever-evolving techniques and approaches, as well as the constantly updated clients. To better understand this complex DHL concept, it is important to try dissecting it accordingly. First and foremost, even though being shown to be a complex and contested concept (1), literacy has been described as one's ability to understand, evaluate, use, and engage with written texts to participate in society, achieve one's goals, and develop one's knowledge and potential (2). Literacy has typically two distinctive domains (3): task-based literacy and skill-based literacy.

Furthermore, the individual's literacy in a health-related context is called health literacy, while being closely related to social determinants of health (education, income, language barriers, and other physical, cultural, and environmental factors) building knowledge and skills related to self-care can mitigate the health impact of low health literacy (4). Then logically DHL derives as the degree to which individuals can obtain, process, and understand basic health information and services needed to make appropriate health decisions. In other means, DHL encompasses the ability to effectively find, interpret, and utilize health-related information from digital sources. The best practices for digital health literacy are more personalized, relevant, interactive, and action-oriented experiences while involving modern measures like artificial intelligence and machine learning, virtual and augmented reality, and blockchain. In a rather systemic context, DHL can move the role of technology beyond data collection to a more integrated system, whereas in a social context, it can help individuals to be transformed from passive to active participants in their own health by being equipped with digital solutions. Lately, DHL has been tipped to be a super determinant of health rather than just the sum of its parts (5), whereas in association with internet connectivity has been also acknowledged as a “super social determinant of health” (6).

In a wide public health context, the failure to equip future professionals with such skills (not just healthcare workers but other care providers like social workers) will most probably facilitate health, social, and economic disparities, undermine patient engagement, and compromise public health initiatives to the core.

Therefore, this article argues for the necessity to integrate DHL courses in every health-related and care-providing profession's curriculum, as a measure to be in line with the ever-evolving global needs.

## 2 DHL education

### 2.1 Digitizing education

If there is one single word that can best describe the modern human it's “digitalization.” The speed of change and evolution observed are without precedent in our thousands of years of history. In a mere 66 years, we have progressed from a prototyped airplane's first flight to a sophisticated spaceship's moon landing. From a 2-song vinyl disc, which served as the primary source of discretionary music until 1962, we have now reached a point where an entire singer‘s oeuvre can be stored on a millimetric thumb drive. Therefore, technology has significantly enriched human life, enhanced the quality, and even increased expected life expectancy at birth. These outcomes are hugely significant, notwithstanding the lack of specific official regulations and recommendations often discarding many novel approaches and digital health technologies (7). Somehow, everything has to keep up with the ever-evolving digitalization of technology. Older generations must adjust to rapid changes, whereas younger generations keep pushing toward novelties. The workforce's time has been significantly shortened and their productivity increased due to the introduction of new technologies. In healthcare, the applications of digital technologies are helping to optimize the diagnosis, consulting, and treatment of patients (7). Healthcare professionals now have more resources to provide in addition to their continual evolution of skills. On the other hand, their valued time could be easily preserved if using technology appropriately to disseminate health-related information. Nowadays, healthcare experts do not necessarily need to be present to provide professional information and can be replaced by recorded videos and other user-friendly tools to guide the end-users.

However, the accelerated pace of technological advancement necessitates that students cultivate the ability to utilize technology in an efficacious manner within their prospective professions. As future experts, their academic training must integrate technology and digital literacy across disciplines. The absence of regulatory frameworks governing emerging technologies underscores the necessity for curricula to adapt in parallel with these innovations.

### 2.2 Curriculum development

The idea of providing the much-needed DHL courses in different educational settings has been emphasized previously (8–11). The COVID-19 pandemic was one of the catalysts of this idea, showing all of us how important this topic is. Utukuri and colleagues (11) argued in their article how health literacy is a neglected part of health curricula. Nonetheless, pervasive inertia appears to persist in addressing this critical deficiency.

The course curriculum would be developed using a systematic approach, including curriculum mapping, stakeholder involvement, and instructional design principles while ensuring alignment with the competencies required by students. This course should have a core concept stratified into 5 main pillars:

1. Governance of health and well-being;
2. Evolution of industrial revolutions;
3. Soft skills in health literacy;
4. Methodologies, strategies, and techniques in health literacy education;
5. Community approach and coaching:

Furthermore, the course needs to be focused on content alignment, student engagement, and practical integration. The content would be developed around these five core pillars, with a specific focus depending on the particular program. The course itself should be introduced, although not exclusively, to students from a variety of academic backgrounds, including medical sciences, allied medical sciences, social workers, and education programs (primary, preschool, or special education). Furthermore, teaching material should take into consideration not only the student's influence on academic performance but also the important inputs of the academic staff that are coherent with the latest developments in the technological sphere. This is because new measurement tools have been developed and used to generate initial data on digital health literacy in educational settings. These tools assess not only the personal capacities of learners and teachers but also of learning opportunities and instructions regarding the teaching and learning of DHL (12). This produces positive effects related to teacher-student communication, course functioning, the development of critical thinking, and the transactional aspect of the curriculum itself.

DHL can play an important role in human well-being and even lifespan. Previous research has indicated that DHL is dependent on several sociodemographic, economic, psychological, and cultural factors (13). Indeed, the benefits of DHL are multidimensional, with the potential to contribute to societal development. Table 1 lists some of the benefits deriving from the increase of DHL.

**Table 1:** The influence of DHL on health.

**DHL influence**	**Increase**	**Decrease**
Prepared workforce	+	
Morbidity		+
Mortality		+
Hospitalization time		+
Chronic health conditions		+
Control over chronic health conditions	+	
Polypharmacy and medication usage		+
Emergency medicine services		+
Overall health and wellbeing	+	
Extensive health-spendings		+

### 2.3 Challenges and barriers

Integration of the DHL as a mandatory course in university entry-level programs within health and care-related disciplines will undoubtedly encounter significant challenges, with resource disparities and technological access being among the most prominent. Perhaps the greatest obstacle in the development of these courses within higher education institutions is the struggle to secure funding for both the development and maintenance of DHL courses, let alone for the needed infrastructure. In such cases, strategic investments from educational and government entities are crucial for the provision and assurance of the essential digital tools and other related resources that are necessary for the implementation of the course. The needed infrastructure and other related means should be provided prior to the course presentation, otherwise, there is a serious risk of the course becoming ineffectual and overly abstract. Then, to ensure a smooth process, the course could be piloted as an elective one in the first year of each program, and then be further integrated as a mandatory course amongst other core subjects.

The lack of skilled experts amongst the faculty on the specific topic represents another significant barrier that necessitates meticulous consideration. Martinez-Ulloa (10) described the risk that the transition to digital health platforms poses for health professionals in their ability to master the use of digital tools. It is important to note that many educators lack professional expertise in digital literacy topics. The absence of qualified or even interested educators to teach such topics not only risks undermining DHL's integration but also might provide resistance among them as they (to some extent understandingly!) could perceive this as a burdensome imposition. The volatile nature of this subject with the rapid technological advancements continuously threatening to outdate knowledge and course material is evident and undeniable. Nevertheless, the modern world is rapidly being digitized and modern infrastructure is continuously being integrated, whereas teaching methods have changed significantly compared to the past. It is imperative that this evolution is followed, or the prospect of perishing looms large. Therefore, educators and institutions must take the initiative to develop their professional skills, expertise, and qualifications in order to equip faculty with the relevant skills and qualifications. This would demonstrate the DHL's integral role within core healthcare and care-providing competencies, which would further advocate for its embedded inclusion across existing curricula.

The emergence of artificial intelligence (AI) and the technologies that facilitate its deployment represents a further transformative factor in the evolution of DHL. AI has the great potential to significantly impact DHL by offering innovative platforms and tools for pedagogical purposes. Such developments could facilitate the creation of novel educational experiences tailored to individual needs. Additionally, AI-powered tools like machine learning and natural language processing can further simplify complex health-related concepts for the end-users. Nonetheless, there are also uncertainties about AI's influence on DHL teaching in health curricula and a close observation needs to be given. This includes the ethical implications of using AI like data privacy, algorithmic bias, or even an ever-increasing reliance on such automated systems.

## 3 Discussion—A call for educational reform

Integration of DHL into curricula of health care and other care-related education is not merely an academic enhancement, but a fundamental necessity in preparing a future-ready care-providing workforce. The incorporation of such courses into the curricula of other educational-related programs, including primary, preschool, and even special education, would undoubtedly equip future educators with the necessary skills and expertise to disseminate knowledge and guidance to the next generation. This approach could potentially serve as a crucial step toward establishing a more secure future, by minimizing the potential impact of misinformation and harmful propaganda.

The widespread implementation of digital literacy competencies in healthcare settings has the potential to empower patients and promote equitable health outcomes. Conversely, failure to act promptly could result in the widening of existing disparities, undermining patient care by exposing an unprepared workforce to the state-of-the-art demands of modern health.

As healthcare continues to undergo significant transformation due to rapid digital advancements, the importance of addressing the associated challenges has never been greater. DHL represents a crucial instrument for bridging knowledge gaps, empowering individuals, and promoting equitable health outcomes. Without immediate integration of DHL into professional training, there is a risk of producing generations of care providers unprepared for the digital demands of modern healthcare, potentially widening existing disparities and undermining the quality and accessibility of patient care.

This article has highlighted the urgent need for comprehensive reforms, with DHL identified as a vital competency for health and other care-related professionals, just as much as for school educators and others alike. By employing targeted curriculum design, optimizing resource allocation, and implementing faculty training, educational institutions can take concrete steps to overcome current barriers and fully actualize the potential of digital health education. Integrating DHL as a staple of health education, not only will practitioners' technical capabilities be enhanced, but also a culture of lifelong learning, adaptability, and ethical responsibility will be fostered, which is vital in our evolving healthcare landscape.

Looking forward, the healthcare sector must adopt the use of DHL as a cornerstone of professional competence, one that not only empowers providers but also actively engages and educates patients. By embedding DHL into health and care curricula, we can shape a new paradigm, one where health professionals and patients alike are equipped with the digital skills to navigate, interpret, and act upon health information with confidence and efficacy. Such a future holds the promise of a more inclusive, informed, and resilient healthcare system, attuned to the needs of a digital age.
